# Editorial: Alternative fertilizer harnessing plant-microbe interactions (AFPMI) for improved soil and plantnutrient management

**DOI:** 10.3389/fpls.2023.1333927

**Published:** 2023-12-06

**Authors:** Adil Mihoub, Iftikhar Ahmad, Emanuele Radicetti

**Affiliations:** ^1^ Center for Scientific and Technical Research on Arid Regions (CRSTRA), Biophysical Environment Station, Touggourt, Algeria; ^2^ Department of Environmental Sciences, COMSATS University Islamabad, Vehari, Pakistan; ^3^ Department of Chemical, Pharmaceutical and Agricultural Sciences (DOCPAS), University of Ferrara, Ferrara, Italy

**Keywords:** nutrient imbalances, bio-fertilizers, beneficial microbes, climate change, field crops, food security, soil health

## Introduction

Worldwide, agriculture is the main source of food for people and is essential to maintaining food security (Jamal et al., 2023). However, there are many challenges in this sector, including the depletion of soil fertility and land degradation. These issues have led to a decrease in the amount of food available per person in various regions globally (Bouma, 2020). The sustainability of agroecosystems, preservation of biodiversity, and food security are threatened by land degradation and climate change (AbdelRahman, 2023; Kumawat et al., 2023). Improper agricultural land management has reduced 25% of total land worldwide and caused an annual soil loss of approximately 24 billion tons (Ejaz et al., 2023). This has serious implication for global food supply because degraded soil is less resilient and less productive (Mihoub et al., 2022a). It is estimated that worldwide food supply is expected to fall by 12% over the next 25 years, resulting in a 30% increase in food costs (AbdelRahman, 2023). A healthy soil that can provide balanced nutrients to plants is essential for increased productivity (Boukhalfa-Deraoui et al., 2015a). Soil is composed of mineral components, organic matter, and microorganisms that have evolved over thousands and millions of years. Unfortunately, due to human activities and improper land management, this delicate balance is disrupted, resulting in rapid soil degradation within a few years (AbdelRahman, 2023).

Climate change and depleting water and land resources have built up pressure on food supply and demand, which will be greatly increased by 2050 to feed 9.7 billion people worldwide (Becker and Fanzo, 2023). Unfortunately, this situation is further worsened by a hike in fertilizer prices and mismanagement of fertilizer allocation (Mihoub and Boukhalfa-Deraoui, 2014). Fertilizer prices have risen significantly in many countries, particularly in Africa, Latin America, and Asia, and smallholder farmers do not have access to fertilizers (Hebebrand and Laborde Debucquet, 2023); thus, rising uncertainty and high fertilizer prices are already affecting food production prospects and the livelihood of farmers (Boukhalfa-Deraoui et al., 2015b; Mihoub et al., 2016). Additional fertilizer production is not the only solution to this crisis, further initiatives must be made to encourage alternative fertilizer sources for sustainable soil management (SSM), sustainable crop production (SCP), and food security in changing climates, which significantly help to support crop production and mitigate the negative impacts of biotic and abiotic stresses (Astapati and Nath, 2023; Kumar et al., 2023). These alternative fertilizer sources play a pivotal role in SSM and SCP, as they not only contribute to cost savings but also offer numerous environmental benefits. By the use of organic fertilizers derived from waste materials not only helps manage and recycle urban waste fluxes but also provides added value in economic terms related to nutrient contents (Khan et al., 2022). Furthermore, the use of bio-fertilizers enriched with beneficial microbes enhances soil health and fertility (Mahmud et al., 2021). Harnessing plant-microbe interactions for alternative fertilization methods is a promising approach to enhance farm productivity and reduce the environmental impact of synthetic chemical fertilizers (Macdonald and Singh, 2014).

The Research Topic, titled “*Alternative Fertilizer Harnessing Plant-Microbe Interactions (AFPMI) for Improved Soil and Plant Nutrient Management,”* explores various strategies to foster alternative fertilizer sources for sustainable soil management, crop production, and food security in changing climates. AFPMI aims to advocate for the use of manures, biochar, bio-fertilizers, nano-fertilizers, and beneficial microbes. The ultimate objective is to maximize crop yields by highlighting the crucial role of alternative fertilizer sources and technologies in soil nutrition. This approach will undoubtedly optimize resource consumption while minimizing detrimental effects on natural resources.

## Alternative fertilizers harnessing plant-microbe interactions and soil health

Improving soil health is crucial with reference to climate change. One effective approach is to use Alternative Fertilizer Harnessing Plant-Microbe Interactions (AFPMI) which combine types of fertilizers like organic, chemical, nano, and bio fertilizers. This holistic method helps in development and proliferation of soil edaphon (Amin and Mihoub, 2021; Daraz et al., 2021; Tahir et al., 2021; Babar et al., 2022), which have a vital role in enhancing soil structure and fertility (Khan et al., 2022; Jamal et al., 2023). By adopting practices that involve Alternative Fertilizers Harnessing Plant-Microbe Interactions, farmers can establish soils that’re better equipped to endure harsh weather conditions and get high economic yields from crops. This resilience is crucial as agriculture faces the challenges posed by climate change. Research conducted by Krasilnikov et al. (2022) further emphasizes the significance of AFPMI in adapting to these climate variations. Through AFPMI practices, farmers can ensure that their soil remains fertile and productive when faced with changing weather patterns. This comprehensive approach not only helps protect the environment but also supports the long-term viability of agricultural systems. As we work towards reducing the effects of climate change, Alternative Fertilizers Harnessing Plant-Microbe Interactions serves as a tool for promoting soil wellbeing and ensuring food production for generations.

## Alternative fertilizer harnessing plant-microbe interactions for improved crop productivity

Alternative Fertilizer Harnessing Plant-Microbe Interactions (AFPMI) for Improved Soil and Plant Nutrient Management plays a vital role in improving plant productivity. This is especially important amid rapid climate change, where ensuring food security is a top priority. AFPMI optimizes crop nutrient utilization, ensuring higher crop yields, and crop resilience through the strategic application of organic and bio-fertilizers (Daraz et al., 2023; Salman et al., 2023; Ullah et al., 2023). By embracing the practices of Alternative Fertilizers Harnessing Plant-Microbe Interactions, we can effectively tackle the challenges presented by climate change and guarantee a sustainable and secure food supply for future generations.

## Alternative fertilizer harnessing plant-microbe interactions: a solution to rising fertilizer cost and food security

Among the major benefits of AFPMI that make it crucial is that it minimizes further reliance on expensive chemical fertilizers. Due to the escalation in fertilizer prices and the consequences of climatic change, compliance with methods brought about by AFPMI indeed emerges as an attractive and cost-effective option for the farming community (Mihoub et al., 2022b; Mihoub et al., 2022c). AFPMI improves the efficiency of nutrient usage and hence reduces the cyclic use of costly chemical inputs. In this way, we can reduce the cost of production and promote sustainable agricultural practices (Khan et al., 2022). Food security is closely attached to the utilization of AFPMI and is especially concerned with challenges linked to climate change. Improved crop yield has been embraced through the adoption of AFPMI practices towards increased production levels required for food security cases (Ahmad et al., 2022; Jamal et al., 2023). AFPMI has tremendous benefits concerning the improvement of soil fertility and acidity vis-à-vis the apparent effects of environmental change such as heavy metals, long droughts, and salt stress (Hamid et al., 2021; Tahir et al., 2021; Ahmad et al., 2022; Daraz et al., 2023). AFPMI techniques are used by farmers to protect food crops from nutrient deficiency, promote uniformity, and predictable food production (Daraz et al., 2021; Hussain et al., 2022). Further, AFPMI is a sustainable kind of agriculture that significantly reduces the impact on the environment. This approach helps in protecting ecosystems and ensuring food security (Nadia et al., 2023), thus fostering development towards a more resilient and safe food future.

## Alternative fertilizer harnessing plant-microbe interactions and climate change adaptation

As the Earth’s climate continues to undergo significant changes, it is crucial to explore effective strategies for adapting to these shifts, particularly in the field of agriculture. AFPMI practices are designed to help crops withstand the increasingly challenging environmental stressors brought about by climate change, such as rising temperatures and unpredictable precipitation patterns. By enhancing the resilience of crops, AFPMI practices enable them to better cope with heat and water scarcity, ultimately safeguarding agricultural productivity (Krasilnikov et al., 2022). AFPMI not only benefits crop directly but also supports global initiatives to mitigate climate change. It contributes to the development of carbon-free agricultural farming, which could be achieved by reducing greenhouse gases in the atmosphere and increasing the passive pool of carbon in the soil through biochar (Wang et al., 2023). Such farming practices would be eco-friendlier and climate resilient.

## Articles and insights

This Research Topic presents a collection of articles that provide insights into the various impacts of alternative fertilizer harnessing plant-microbe interactions (AFPMI) on sustainable agriculture. These research contributions cover important aspects of agricultural sustainability, including interventions in soil microbiota and the management of organic waste. This editorial outline the significant findings of all the manuscript published in this Research Topic with a focus on their significance to soil and plant health, crop yields, fertilizer costs, food security, and climate change. One of the articles discusses the long-term application of sheep manure fertilizer at a depth of 50–70 cm on tea plantations. This practice has been found to alleviate soil acidification by improving soil pH, ammonium nitrogen content, nitrogen fixation ability, and root activity. As a result, tea tree roots are better able to absorb nitrogen from the soil, leading to increased tea yield and quality (Jia et al.). This research not only contributes to sustainable agriculture but also reduces the need for chemical pesticides and promotes soil health.

Biofertilizer or PGPR has a vital role in maintaining soil and plant health thus, five studies deal with either the use of sole biofertilizer or its co-inoculation with organic fertilizers. For instance, Ma et al. isolated the new bacterial strain MQR6T (*Pantoea rhizosphaerae*) from the rhizosphere of *Acer truncatum* Bunge. This strain can solubilize inorganic P produce indole acetic acid and siderophores. The inoculation of strain MQR6T with *A. truncatum* improves plant growth, biomass, and P-accumulation in plants. This strategy has the potential to reduce dependence on inorganic fertilizers, improve benefit to cost ratio, thus contribute to food security. This study advocates the use of blend of biofertilizers, organic fertilizers, and chemical fertilizers to improve wheat yield. The combination of DAP+FYM+BA treatment not only improves wheat growth, biomass, and yield but also improves the quality of wheat grain (Asghar et al.).

This research highlights the positive role of biofertilizers supplements as alternatives to conventional fertilizers to promote soil health, increase productivity, and food security while reducing the ecological footprint. This study reveals that the application of biofertilizers can help the strawberry plant overcome the negative effects of nitrogen deficiency (García-López et al.). PGPR-inoculation improved the economic yield of strawberries by enhancing the efficiency of photosystem-II and N-nutrition for plants in an N-deficient environment. This approach confirms that biofertilizers may be promising complementary tools to improve nitrogen-based chemical fertilization processes, thereby significantly reducing the use of this agrochemical. This study (Ujvári et al.) reveals that microbial communities regulate soil fertility and are mainly dependent on crop species and cropping systems adapted by farmers (Ujvári et al.). The NP fertilization and maize genotypes played a vital role in reshaping the microbial communities in the maize rhizosphere at the plant emergence stage. Application of NP fertilization, maize hybrids, and biostimulants increased plant growth, early flowering, and the economic yield of maize in field conditions (Capo et al.). They also proposed alternative techniques, such as utilizing early-vigor genotypes or applying biostimulants to seeds, which can help reduce the negative impacts of abiotic stresses associated with early planting and result in faster crop development. These innovations may minimize or, in some circumstances, eliminate the need for starting fertilization.

Two studies focus on the application of pristine biochar or its combination with fishpond sediment and iron as an alternative or supplement source of nutrient management under nutrient or heavy metal stress. Mahmood et al. highlighted the significance of using the optimal amounts of biochar and fishpond sediments to improve the nutrient content and availability of soil. It provides valuable information on how biochar and fishpond sediments can be used together to reduce phosphorus fixation and prevent the loss of residual phosphorus. Ultimately, this approach helps to sustain food production. In another study, Algethami et al. found that application of iron-modified biochar (Fe-BC) improved the nutritional quality of soil and mitigated metal toxicity in wheat plants under heavy metal stress. The results showed that Fe-BC is a promising strategy for improving soil fertility and reducing the harmful effects of Cd and Pb contamination. This research has significant implications for enhancing food security in stressful environments.

## Concluding remarks and future perspectives

Alternative fertilizer harnessing plant-microbe interactions (AFPMI) play a vital role in improving soil health, plant productivity, and climate resilience. We can achieve maximum economic yields through judicious application of organic fertilizers (sheep manure, biochar), microbial-based fertilizers (biofertilizers), synergistic use of bio-synthetic fertilizers (PGPR + chemical fertilizers), adopting various agronomic practices (seed priming, crop genotypes) and cropping systems. These AFPMI practices not only improve soil fertility status and soil edaphon but also translate it into better crop stand, economic yields, and resistance to climate change. By utilizing agricultural technologies and adopting innovative practices, we have the potential to create a future where sustainable agriculture forms the basis of food security, despite the challenges posed by climate change.

## Author contributions

AM: Conceptualization, Validation, Visualization, Writing – original draft, Writing – review & editing. IA: Writing – original draft, Writing – review & editing. ER: Writing – original draft, Writing – review & editing.

## References

[B1] AbdelRahman M. A. E. (2023). An overview of land degradation, desertification and sustainable land management using GIS and remote sensing applications. Rendiconti Lincei. Sci. Fisiche e Naturali 34, 1–42. doi: 10.1007/s12210-023-01155-3

[B2] Ahmad I. Munsif F. Mihoub A. Jamal A. Saeed M. F. Babar S.. (2022). Beneficial effect of melatonin on growth and chlorophyll content in wheat (*Triticum aestivum* L.) grown under salt stress conditions. Gesunde Pflanzen 74 (4), 997–1009. doi: 10.1007/s10343-022-00684-5

[B3] Amin A.E.E.A.Z. Mihoub A. (2021). Effect of sulfur-enriched biochar in combination with sulfur-oxidizing bacterium (*Thiobacillus* spp.) on release and distribution of phosphorus in high calcareous p-fixing soils. J. Soil Sci. Plant Nutr. 21 (3), 2041–2047. doi: 10.1007/s42729-021-00500-5

[B4] Astapati A. D. Nath S. (2023). The complex interplay between plant-microbe and virus interactions in sustainable agriculture: Harnessing phytomicrobiomes for enhanced soil health, designer plants, resource use efficiency, and food security. Crop Design 2, 100028. doi: 10.1016/j.cropd.2023.100028

[B5] Babar S. Jilani G. Mihoub A. Jamal A. Ahmad I. Chaudhary A. N.. (2022). Bacterial redox cycling of manganese in calcareous soil enhances the nutrients bioavailability to wheat. J. Soil Sci. Plant Nutr. 21 (3), 1215–1223. doi: 10.1007/s42729-021-00725-4

[B6] Becker S. Fanzo J. (2023). Population and food systems: what does the future hold? Population Environ. 45 (3), 20. doi: 10.1007/s11111-023-00431-6

[B8] Boukhalfa-Deraoui N. Hanifi-Mekliche L. Mekliche A. Mihoub A. Daddibouhoun M. (2015b). Effect of phosphorus application on durum wheat in alkaline sandy soil in arid condition of Southern Algeria. Asian J. Crop Sci. 7 (1), 61–71. doi: 10.3923/ajcs.2015.61.71

[B7] Boukhalfa-Deraoui N. Hanifi-Mekliche L. Mihoub A. (2015a). Effect of incubation period of phosphorus fertilizer on some properties of sandy soil with low calcareous content, Southern Algeria. Asian J. Agric. Res. 9 (3), 123–131. doi: 10.3923/ajar.2015.123.131

[B9] Bouma J. (2020). Soil security as a roadmap focusing soil contributions on sustainable development agendas. Soil Secur. 1, 100001. doi: 10.1016/j.soisec.2020.100001

[B10] Daraz U. Ahmad I. Li Q. S. Zhu B. Saeed M. F. Li Y.. (2023). Plant growth promoting rhizobacteria induced metal and salt stress tolerance in *Brassica juncea* through ion homeostasis. Ecotoxicology Environ. Saf. 267, 115657. doi: 10.1016/j.ecoenv.2023.115657 37924800

[B11] Daraz U. Li Y. Sun Q. Zhang M. Ahmad I. (2021). Inoculation of Bacillus spp. modulate the soil bacterial communities and available nutrients in the rhizosphere of vetiver plant irrigated with acid mine drainage. Chemosphere 263, 128345. doi: 10.1016/j.chemosphere.2020.128345 33297270

[B12] Ejaz N. Elhag M. Bahrawi J. Zhang L. Gabriel H. F. Rahman K. U. (2023). Soil erosion modelling and accumulation using RUSLE and remote sensing techniques: case study Wadi Baysh, Kingdom of Saudi Arabia. Sustainability 15 (4), 3218. doi: 10.3390/su15043218

[B13] Hamid B. Zaman M. Farooq S. Fatima S. Sayyed R. Z. Baba Z. A.. (2021). Bacterial plant biostimulants: a sustainable way towards improving growth, productivity, and health of crops. Sustainability 13 (5), 2856. doi: 10.3390/su13052856

[B14] Hebebrand C. Laborde Debucquet D. (2023). “High fertilizer prices contribute to rising global food security concerns,” in The Russia-Ukraine conflict and global food security, (International Food Policy Research Institute (IFPRI)), 7, 38–42.

[B15] Hussain S. Khan S. M. Jamal A. Mihoub A. Saeed M. F. Khalid M. S.. (2022). Improvement of growth, yield and biochemical properties of potato (*Solanum tuberosum* L.”Montreal” and “Red bull”) by foliar application of zinc under calcareous soil conditions. Gesunde Pflanzen 74 (3), 561–570. doi: 10.1007/s10343-022-00631-4

[B16] Jamal A. Saeed M. F. Mihoub A. Hopkins B. G. Ahmad I. Naeem A. (2023). Integrated use of phosphorus fertilizer and farmyard manure improves wheat productivity by improving soil quality and P availability in calcareous soil under subhumid conditions. Front. Plant Sci. 14. doi: 10.3389/fpls.2023.1034421 PMC9900179 36755699

[B17] Khan I., Amanullah Jamal A. Mihoub A. Farooq O. Farhan Saeed M.. (2022). Partial substitution of chemical fertilizers with organic supplements increased wheat productivity and profitability under limited and assured irrigation regimes. Agriculture 12 (11), 1754. doi: 10.3390/agriculture12111754

[B18] Krasilnikov P. Taboada M. A. Amanullah (2022). Fertilizer use, soil health and agricultural sustainability. Agriculture 12 (4), 462. doi: 10.3390/agriculture12040462

[B19] Kumar U. Raj S. Sreenikethanam A. Maddheshiya R. Kumari S. Han S.. (2023). Multi-omics approaches in plant–microbe interactions hold enormous promise for sustainable agriculture. Agronomy 13 (7), 1804. doi: 10.3390/agronomy13071804

[B20] Kumawat A. Yadav D. Srivastava P. Babu S. Kumar D. Singh D.. (2023). Restoration of agroecosystems with conservation agriculture for food security to achieve sustainable development goals. Land Degradation Dev. 34, 3079–3097. doi: 10.1002/ldr.4677

[B21] Macdonald C. Singh B. (2014). Harnessing plant-microbe interactions for enhancing farm productivity. Bioengineered 5 (1), 5–9. doi: 10.4161/bioe.25320 23799872 PMC4008467

[B22] Mahmud A. A. Upadhyay S. K. Srivastava A. K. Bhojiya A. A. (2021). Biofertilizers: A Nexus between soil fertility and crop productivity under abiotic stress. Curr. Res. Environ. Sustainability 3, 100063. doi: 10.1016/j.crsust.2021.100063

[B24] Mihoub A. Amin A.E.E.A.Z. Motaghian H. R. Saeed M. F. Naeem A. (2022b). Citric acid (CA)–modified biochar improved available phosphorus concentration and its half-life in a P-fertilized calcareous sandy soil. J. Soil Sci. Plant Nutr. 21, 465–474. doi: 10.1007/s42729-021-00662-2

[B23] Mihoub A. Boukhalfa-Deraoui N. (2014). Performance of different phosphorus fertilizer types on wheat grown in calcareous sandy soil of El-Menia, southern Algeria. Asian J. Crop Sci. 6 (4), 383–391. doi: 10.3923/ajcs.2014.383.391

[B25] Mihoub A. Daddi Bouhoun M. Saker M. L. (2016). Phosphorus adsorption isotherm: A key aspect for effective use and environmentally friendly management of phosphorus fertilizers in calcareous soils. Commun. Soil Sci. Plant Anal. 47 (16), 1920–1929. doi: 10.1080/00103624.2016.1206923

[B26] Mihoub A. Koull N. Helimi S. Elhafed Kherraze M. Mokhtari S. Pulido Fernández M. (2022a). Developing scoring functions for soil quality to assess land suitability for irrigated wheat in Southern Algeria. Soil Use Manage. 38 (1), 262–276. doi: 10.1111/sum.12770

[B27] Mihoub A. Naeem A. Amin A.E.E.A.Z. Jamal A. Saeed M. F. (2022c). Pigeon manure tea improves phosphorus availability and wheat growth through decreasing p adsorption in a calcareous sandy soil. Commun. Soil Sci. Plant Anal. 53 (19), 2596–2607. doi: 10.1080/00103624.2022.2072859

[B28] Nadia Amanullah Arif M. Muhammad D. (2023). Improvement in wheat productivity with integrated management of beneficial microbes along with organic and inorganic phosphorus sources. Agriculture 13 (6), 1118. doi: 10.3390/agriculture13061118

[B29] Salman M. Inamullah Jamal A. Mihoub A. Saeed M. F. Radicetti E.. (2023). Composting sugarcane filter mud with different sources differently benefits sweet maize. Agronomy 13 (3), 748. doi: 10.3390/agronomy13030748

[B30] Tahir M. Imran M. Nawaz F. Shahid M. Naeem M. A. Ahmad I.. (2021). Effects of Bacillus sp. MR-1/2 and magnetite nanoparticles on yield improvement of rice by urea fertilizer under different watering regimes. J. Appl. Microbiol. 131 (5), 2433–2447. doi: 10.1111/jam.15110 33896080

[B31] Ullah J. Shah S. Mihoub A. Jamal A. Saeed M. F. Székely Á.. (2023). Assessing the effect of combining phosphorus fertilizers with crop residues on maize (*Zea Mays* L.) productivity and financial benefits. Gesunde Pflanzen 75, 1995–2008. doi: 10.1007/s10343-023-00829-0

[B32] Wang L. Chen D. Zhu L. (2023). Biochar carbon sequestration potential rectification in soils: Synthesis effects of biochar on soil CO_2_, CH_4_ and N_2_O emissions. Sci. Total Environ. 904, 167047. doi: 10.1016/j.scitotenv.2023.167047 37716679

